# Thoracolumbar myelopathies in pug dogs

**DOI:** 10.1111/jvim.16639

**Published:** 2023-02-06

**Authors:** Ian J. Wachowiak, Jon S. Patterson, Kathryn M. Winger, Kathleen L. Smiler, Robert Cole, Rachel Moon, Michael Kluz, Lisa R. Bartner

**Affiliations:** ^1^ Department of Small Animal Clinical Sciences Colorado State University Fort Collins Colorado USA; ^2^ Department of Pathobiology and Diagnostic Investigation Michigan State University East Lansing Michigan USA; ^3^ Department of Small Animal Clinical Sciences Michigan State University East Lansing Michigan USA; ^4^ ACLAM Michigan State University College of Veterinary Medicine East Lansing Michigan USA; ^5^ Department of Small Animal Clinical Sciences Auburn University Auburn Alabama USA; ^6^ VCA Jackson Michigan Animal Hospital Jackson Michigan USA

**Keywords:** chronic, constrictive myelopathy, meningeal fibrosis, thoracolumbar arachnoid fibrosis

## Abstract

**Background:**

Constrictive myelopathy (CM) involving a fibrous band around the spinal cord is a newly recognized disease in pug dogs.

**Objectives:**

To identify the frequency of CM based on diagnostic imaging supplemented with necropsy; to determine whether a relationship exists between the sites of CM and other described T3‐L3 myelopathies; and to determine the frequency of caudal articular process dysplasia (CAPD).

**Animals:**

Thirty‐two client‐owned pug dogs diagnosed with a chronic, progressive T3‐L3 myelopathy based on neurological examination performed by a board‐certified neurologist.

**Methods:**

This is a prospective study. All dogs underwent computed tomography (CT) and magnetic resonance imaging (MRI) reviewed by a board‐certified radiologist. Magnetic resonance imaging abnormalities were categorized into diseases; CM only, CM plus other non‐CM condition(s), or non‐CM condition. Sites of CAPD were reported on CT. Nineteen dogs underwent necropsy.

**Results:**

Magnetic resonance imaging revealed 3 dogs with CM only, 17 with CM plus at least 1 other myelopathy, 11 dogs with non‐CM myelopathies only, and 1 with no MRI abnormalities. Nineteen of 32 dogs had >1 myelopathy diagnosis on MRI whereas 15/32 had >1 site of spinal cord compression. All dogs had CAPD at >1 site in the T3‐L3 vertebral column on CT.

**Conclusions and Clinical Importance:**

Constrictive myelopathy affected more than half of pug dogs presenting with chronic thoracolumbar myelopathies. Most had multilevel disease, concurrent myelopathies, or both. There was no apparent relationship between anatomic locations of CAPD and most severe myelopathy or myelopathy type.

**Abbreviations**
CAPD: caudal articular process dysplasia
CM: constrictive myelopathy
CT: computed tomography
IVDH: intervertebral disc herniation
MRI: magnetic resonance imaging
SAD: spinal arachnoid diverticulum

## 1 INTRODUCTION

Chronic, progressive thoracolumbar (TL) myelopathies are common neurological problems in pug dogs, and multiple abnormalities have been described affecting the TL spinal cord and vertebral column in this breed.^1^ Constrictive myelopathy (CM) was first described in pug dogs in 2013^2^ and in a 2014 report, pug dogs were cited as the most commonly affected breed with spinal arachnoid diverticulum (SAD) in a study of 122 dogs.^3^ Both conditions have been associated with spinal cord compression and meningeal (arachnoid, dural, or both) fibrosis,^4^, ^5^ with a constrictive, circumferential band of connective tissue forming in CM and a cerebrospinal fluid‐filled, subarachnoid cavity forming in SAD. This could suggest an underlying relationship in the pathogeneses of these conditions. Intervertebral disc herniations (IVDH) are well described in chondrodystrophic and nonchondrodystrophic breeds,^6^, ^7^ and caudal articular process dysplasia (CAPD) and hemivertebrae are examples of congenital vertebral column disorders that might not only affect the spinal cord, but which are especially common in pug dogs.^8^, ^9^, ^10^ Instability associated with these vertebral malformations has been hypothesized as a reason for development of myelopathy. For example, instability associated with CAPD might lead to CM, which develops gradually over time as a result of chronic micromotion, leading to meningeal fibrosis.^9^, ^11^ The fibrous adhesions could also disrupt normal CSF flow, possibly leading to SAD formation.^9^ On the other hand, a high percentage (97.0%) of neurologically normal pug dogs in 1 study had CAPD.^12^ In another, among 16 pug dogs with CAPD, 5 had no neurological dysfunction.^2^ In the 11 dogs with chronic myelopathies, thickened fibrous meninges encompassed the spinal cord in the regions of CAPD, suggesting an association between the vertebral malformation and CM.

A relationship between meningeal thickening by fibrous connective tissue surrounding the spinal cord resulting in neurological dysfunction associated with CAPD was first proposed in 2013 in a condition termed “constrictive myelopathy” which appeared to be unique to pug dogs.^2^ Since then, many other names have been used, including “pug myelopathy,” “thoracolumbar myelopathy,” “meningeal fibrosis,” and “thoracolumbar arachnoid fibrosis.”^5^, ^10^, ^13^ In this report, the authors will use the term constrictive myelopathy to refer to this condition. Despite recent published reports, the pathogenesis, prevalence, and potential relationship of CM with other chronic TL myelopathies such as SAD and IVDH, and with vertebral malformations such as CAPD remain uncertain.

The relatively common occurrence of various types of chronic TL myelopathies in pug dogs, as well as controversy regarding the role of CAPD in these myelopathies, warrant further investigation. Previous studies have not investigated imaging and postmortem findings simultaneously, and many studies excluded conditions such as IVDH or SAD.

The main purpose of this study was to characterize the diagnosis associated with chronic TL myelopathies in pug dogs and to identify the frequency of CM with and without concurrent myelopathies based on diagnostic imaging. Where available, pathological findings are included. A secondary aim was to determine whether a relationship exists between the sites of CM and other described T3‐L3 myelopathies (such as IVDH and SAD) and the sites and frequency of CAPD.

## 2 MATERIALS AND METHODS

Thirty‐two client‐owned pug dogs examined prospectively by a board‐certified veterinary neurologist at Michigan State University (MSU) Veterinary Medical Center (VMC) were included in the study between April 2014 and March 2016. The study was approved by the MSU Institutional Animal Care and Use Committee before dog enrollment.

### 2.1 Inclusion criteria

Pug dogs were included in this prospective study if history and neurological examination indicated a chronic (>7 days) progressive T3‐L3 myelopathy with no relevant abnormalities revealed by complete blood count and serum biochemistry. A T3‐L3 myelopathy was defined as paraparesis of any severity, normal to increased tone and reflexes in the pelvic limbs, with normal thoracic limbs. Consent to postmortem examination was not a requirement for enrollment in this study. Six‐month follow‐up recheck visits were advised, although if distance from MSU was a hindrance, information was gleaned through contact with dogs' local veterinarians.

### 2.2 Exclusion criteria

Pug dogs were excluded from the study if neurological examination localized issues to any region of the nervous system other than, or in addition to the T3‐L3 spinal cord, or if clinical duration of neurological disease was less than 7 days. Dogs also were excluded if noteworthy non‐neurological comorbidities were found.

### 2.3 Study cohort

Pug dog websites and social media managed by 1 author (K. Smiler) were used for recruitment of most study dogs. Eligibility was determined upon neurological examination and review of bloodwork by 1 author (K. Winger). Signalment and clinical information collected for all dogs included age at onset, characteristics of initial signs and clinical progression, presence of fecal or urinary incontinence, and signs of pain. All dogs underwent diagnostic imaging studies under general anesthesia consisting of dexmedetomidine as a premedication, propofol for induction, and sevoflurane as the inhalant anesthetic. Antisedan was used to reverse the dexmedetomidine, if needed. Dogs that were euthanized or died naturally were submitted for necropsy only with the owner's consent.

### 2.4 Diagnostic imaging

Enrolled dogs underwent both computed tomography (CT) and magnetic resonance imaging (MRI) to examine the TL vertebral articular processes and spinal cord. The CT was performed using a helical 16‐slice scanner (GE Brightspeed, General Electric Company, Milwaukee, Wisconsin), with the animal in dorsal recumbency under general anesthesia. Parameters for the scan included a slice thickness of 0.625 mm, collimator pitch of 1, table speed of 13.75 mm/s, tube rotation time of 1 revolution/s, kV of 120, and mA of 250. Images were then constructed with application of a high‐ and low‐pass algorithm for inspection of bone and soft tissue structures, respectively, without contrast. Computed tomography images allowed for high‐pass algorithm DICOM images to be further reconstructed with Voxar 3D software (Toshiba Medical Visualization Systems Europe Ltd., Edinburgh, UK), for close inspection of the vertebral articular processes for malformation or absence.

The MRI was performed with the dog in dorsal recumbency under general anesthesia, on a 1.5‐T Siemens Espree, with a 4‐channel flexible body coil placed ventrally on the dog. Standard sequences of the TL spine with T1‐ and T2‐weighted images were performed in sagittal and transverse planes, as well as less standard sequences of FLASH (a heavily T2‐weighted sagittal sequence highlighting CSF signal), sagittal and dorsal plane STIR sequences. Three‐plane (sagittal, transverse, and dorsal) postintravenous gadoteridol (ProHance, Bracco, 0.2 mmol/kg IV) contrast administration T1‐weighted images were performed if suspicion of a neoplastic or inflammatory lesion was seen by the radiologist during initial sequence evaluation, while the scan was taking place.

All CT and MRI images were visually inspected and analyzed on DICOM viewing software integrated with the PACS system, Horizon Rad Station (McKesson, San Francisco, California).

One board‐certified radiologist interpreted all CT scans. All MRI studies were interpreted by 2 board‐certified radiologists at Auburn University College of Veterinary Medicine (R. Cole and R. Moon). A consensus was reached between both readers. A diagnosis of CAPD was based on CT scans by noting small or absent caudal articular processes with suboptimal articulation with the cranial articular process of the next caudal vertebra. Other vertebral malformations such as wedge or butterfly vertebrae were recorded but not investigated further for the purposes of this study. Caudal articular process hypoplasia or aplasia was reported as dysplasia. The affected sites and laterality were also recorded. The locations of CAPD on CT were compared to locations of TL lesions diagnosed on MRI^13^ by recording locations of both osseous and spinal cord abnormalities and noting association by vicinity.

The sites and types of spinal cord lesions, collectively referred to here as *myelopathies*, were determined by MRI studies. Myelopathies were categorized as CM only, CM plus at least 1 other non‐CM condition, or non‐CM condition. Non‐CM diagnoses included IVDH and SAD (other non‐CM diagnoses, such as neoplasia and trauma, were considered but these diagnoses were not apparent by MRI among study dogs). Normal spinal cord (no abnormalities detected by MRI) was an additional category.

**FIGURE 1:**
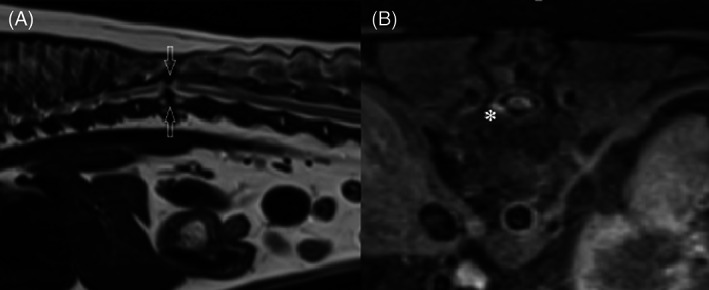
(A) Sagittal T2‐weighted magnetic resonance (MR) image of the thoracolumbar region displaying a hypointense ventral and dorsal spinal cord compression and focal spinal cord atrophy at the level of T12‐T13 indicated by the arrows. (B) Transverse T1‐weighted fat saturated postcontrast MR image of the same dog at level of T12 displaying a hypointense band of tissue predominantly dorsolateral to the spinal cord. There is contrast enhancement of the ventrolateral meninges and spinal cord atrophy at this location indicated by the asterisk.

Magnetic resonance imaging criteria for CM included the presence of T1‐ and T2‐weighted hypointense bands of tissue located dorsal or dorsolateral to the spinal cord, focal widening, irregular margins, and T2‐weighted hypointense or heterogeneous signal intensity of the dorsal subarachnoid space and associated spinal cord compression or atrophy^14^ (Figure 1).

Intervertebral disc herniation was diagnosed when there was loss of the normal T2‐weighted signal intensity of the nucleus pulposus, narrowing of the intervertebral disc space, and intervertebral disc protrusion or extrusion of disc material into the vertebral canal. Protrusions and extrusions were not differentiated on MRI and were grouped as herniations. In general, lesions were deemed compressive by 1 or more of the following findings: displacement of surrounding CSF and epidural fat, presence of extradural material consistent with disc material (typically T1‐ and T2‐weighted hypointense) causing a mass effect including protrusion or extrusion, altered shape of the spinal cord, and displacement of the spinal cord within the vertebral canal.^6^, ^7^

Magnetic resonance imaging criteria for SAD included a focal dilation of the subarachnoid space with CSF resulting in intradural, extramedullary compression of the adjacent spinal cord. This was best appreciated on T2‐weighted images where the CSF was hyperintense. In studies that included pre‐ and postcontrast T1‐weighted images, the focal dilation of the subarachnoid space was hypointense and noncontrast enhancing, as described.^15^

When multiple lesions, sites of compression, or both were present within the same individual, all present myelopathies were noted and that dog was placed in a multiple diagnoses category; in addition, the prominent diagnosis was selected based on the greatest degree of spinal cord disease. Degree of compression was not noted as it was assumed that all compressive lesions could have contributed to the clinical myelopathy.

### 2.5 Pathology

Postmortem examination was performed by a board‐certified veterinary pathologist (JP) if the dog was brought to MSU after euthanasia or natural death. A dorsal laminectomy was completed from the first cervical (C1) vertebra through the sacrum to expose the entire spinal cord. The brain was removed in whole from the cranial cavity. The vertebral column containing the spinal cord and the brain were submersed in 10% neutral‐buffered formalin for 48 hours. After complete fixation, the spinal cord was removed from the vertebral canal, photographed, and sectioned. The spinal cord was first divided into transverse sections at the level of each intervertebral disc. Next, parasagittal dorsoventral sections were made through the spinal cord segments so that the dorsal and ventral gray matter horns were included. The entire spinal cord was included for histopathological examination. All sections were routinely processed and stained with hematoxylin and eosin (H&E).

Representative sections of the brain (cerebrum, cerebellum, thalamus, midbrain, pons, and medulla) were also included. If gross lesions were observed, additional sections of brain were submitted for histopathological examination.

A postmortem diagnosis of CM was based on gross or microscopic segmental meningeal thickening due to arachnoid fibrosis over the dorsal half of the spinal cord or circumferentially. Intervertebral disc herniation was grossly defined by loss of distinction between the nucleus pulposus and annulus fibrosus, a mottled opaque gray and brown or white (suggesting calcification) disc with a flaky or friable texture, and dorsal herniation of the disc, causing grossly visible spinal cord compression. Spinal arachnoid diverticulum was diagnosed at necropsy based on finding a subdural “bubble” with compression of the underlying spinal cord seen on transverse sectioning.

### 2.6 Data analysis

Demographic data and data related to clinical signs were reported as medians, ranges, proportions, or percentages, as appropriate. Data related to MRI diagnostic categories (CM, non‐CM only, CM plus at least 1 non‐CM diagnosis) were compared similarly. Frequency and locations of CAPD were summarized in table format, and relationships of MRI diagnostic categories and myelopathy locations to CAPD were simply described. Necropsy findings were reported in relation to diagnostic imaging findings.

## 3 RESULTS

### 3.1 Study cohort

Among the 32 pug dogs in the study, 17 dogs (53%) were neutered females, 14 dogs (44%) were neutered males, and 1 (3%) was an intact female. The median age of onset of neurological clinical signs was 9.0 years, (range, 4.2‐13.1 years). The median interval between onset of neurological signs reported by owners and initial presentation at MSU VMC was 7 months (range, 0.3 months [10 days] to 36 months).

On initial neurological evaluation all dogs demonstrated neurological dysfunction of their pelvic limbs, sparing the thoracic limbs, suggestive of a chronic, progressive TL myelopathy. All dogs were paraparetic with general proprioceptive ataxia; 29 dogs (90%) were ambulatory paraparetic and 3 dogs (10%) were nonambulatory paraparetic often with asymmetric neurological deficits. None of the pug dogs displayed signs of hyperesthesia within the TL region. All dogs had progression of pelvic limb neurological deficits, whereas only 2 dogs (6%) progressed from paraparesis to paraplegia within the timeframe of this study; in these dogs, paraplegia developed at 5 and 8 months after initial onset of clinical signs.

At time of presentation to MSU, urinary incontinence was reported in 1 dog (3%) and fecal incontinence in 4 dogs (12.5%). During the study period, 5 (15.5%) additional dogs developed urinary incontinence and 14 (44%) additional dogs developed fecal incontinence. The median time interval after onset of pelvic limb dysfunction in dogs that developed fecal incontinence after initial presentation was 7 months (range, 1‐35 months), while the median interval after onset of signs of neurologic disease in dogs that developed urinary incontinence after initial presentation was also 7 months (range, 3‐21 months). One dog developed both fecal and urinary incontinence 12 months after onset of paraparesis, and 1 developed both 18 months after onset of paraparesis.

Twenty‐five of the 32 pug dogs (78%) were euthanized or died naturally between the initial visit to MSU and March 2019. Seventeen of the 25 deceased dogs (68%) underwent a nervous system focused necropsy. Sixteen of the 17 dogs (94%) submitted for necropsy were euthanized because of progression of paraparesis and incontinence related to their myelopathies, and 1 dog died naturally (partly from complications associated with diabetes mellitus). Among the 8 dogs known to have died without necropsy, 3 were reportedly euthanized due to severe, progressive myelopathy, 2 were euthanized due to development of other conditions (seizure disorder, lymphoma), and 3 died naturally or were euthanized for unknown reasons. The median age at the time of death for the 25 dogs which were euthanized or died naturally was 11.3 years (range, 6.7‐15.4 years). The median time interval between onset of neurological gait abnormalities and death for these dogs was 23.9 months (range, 0.6‐84.3 months). Seven dogs (22%) were either known to be alive at the time of completion of the study or had been lost to follow‐up.

### 3.2 Diagnostic imaging

Thirty‐two pug dogs received a CT without contrast. All pug dogs in this study were diagnosed with vertebral CAPD and all lesions occurred between the T1‐T2 and the last T‐L1 intervertebral articulations. Thirty‐two of 32 dogs (100%) had CAPD involving T3‐T7 vertebrae, 31/32 (96.9%) had CAPD at T2, T8, and T9 vertebrae, and 23‐30/32 had CAPD between T10 and T12 (Figure 4). Among the 26 thoracic intervertebral articulations (13 right and 13 left side) evaluated in the 32 study dogs as a group, only 55 CAPD lesions were unilateral, with 40 unilateral lesions on the right side and 15 on the left side. Considering the 26 thoracic intervertebral articulation sites in individual dogs, the fewest number of articular processes affected in an individual dog was 12, and the most were 25 processes. Only 2/32 (6.25%) had CAPD between L1 and L3 (both at L2‐L3; Figure 4.).

**FIGURE 2:**
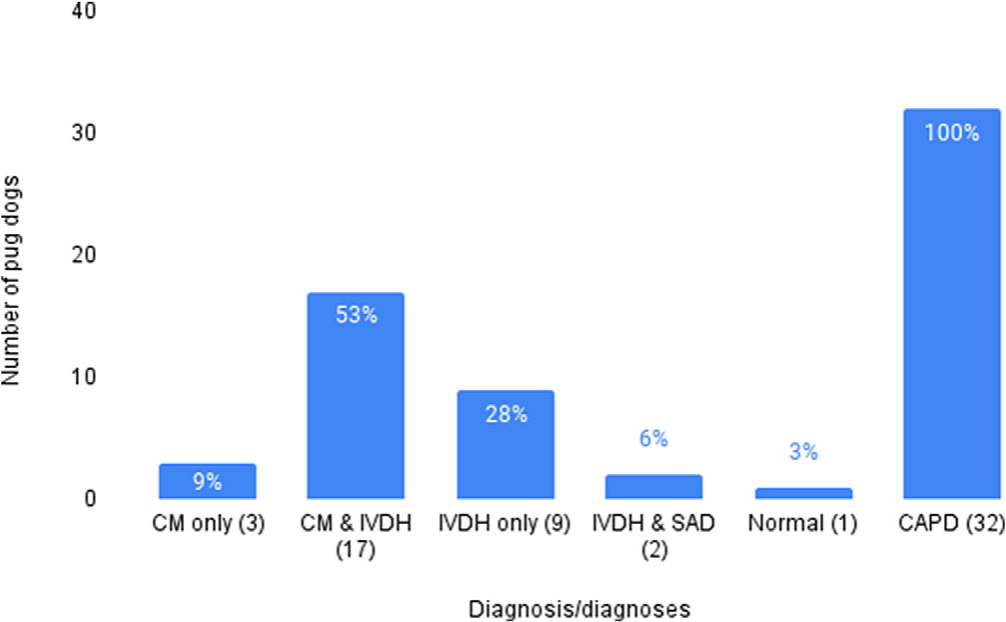
Bar graph representing total number of each myelopathy present in all pug dogs in this study. While caudal articular process dysplasia (CAPD) was present in all studied dogs, constrictive myelopathy (CM) and intervertebral disc herniation (IVDH) were the most frequently encountered compressive myelopathy on neuroimaging. No dogs had CM and subarachnoid diverticulum (SAD) combined, nor SAD alone.

All 32 dogs underwent an MRI in which 8/32 (25%) had contrast studies. The most common diagnosis category in our studied sample was CM accompanied by IVDH (Figure 2). Among 20 dogs diagnosed with CM, 3 dogs were diagnosed with CM only, whereas 17 had CM and IVDH. No dogs had CM and SAD combined. Constrictive myelopathy was noted between vertebral segments T10‐T13 and most commonly diagnosed at vertebral segment T12‐T13. All pug dogs had CAPD whereas the diagnosis of CM alone was noted in 3/32 (9.4%).

Among the dogs with the non‐CM diagnoses only, there were 9 with only IVDH, at single or multiple sites. Intervertebral disc herniation was noted between vertebral segments T11‐L2, most commonly at T11‐T12. Two dogs had SAD between vertebral segments T10‐T12 accompanied by IVDH. One dog had CAPD present with a normal spinal cord based upon MRI review. No dogs had SAD alone.

**FIGURE 3:**
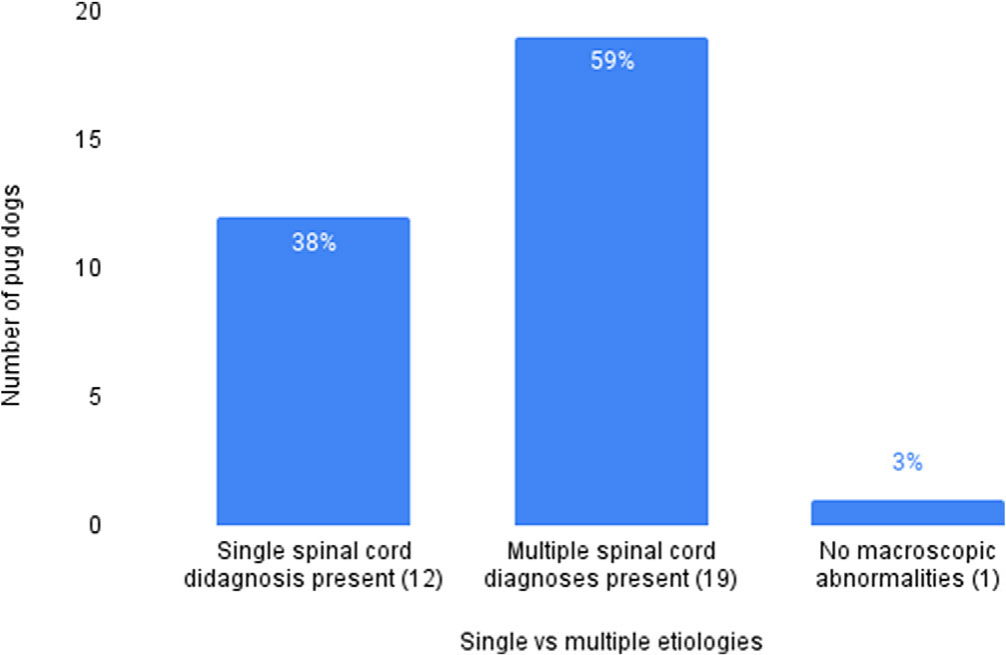
Bar graph displaying number of pug dogs diagnosed with 1 type of compressive spinal cord disease or multiple types of disease. There was 1 dog diagnosed with no spinal cord lesions visible on magnetic resonance imaging. Caudal articular process dysplasia was present in all dogs and is not presented in this figure.

Pug dogs were more likely to have multiple concurrent myelopathies than a single myelopathy (Figure 3). Based on MRI findings, there were 12/32 (37.5%) dogs with 1 type of spinal cord lesion, 19/32 (59.4%) dogs with multiple lesion types, and 1/32 (3.1%) with no lesions noted. Nine of 32 dogs (28.1%) had 1 site of spinal cord compression whereas 23/32 (71.9%) had multifocal compressive myelopathies. No initial or subsequent clinical signs were distinctive as to favor 1 myelopathic diagnosis or category over another.

**FIGURE 4:**
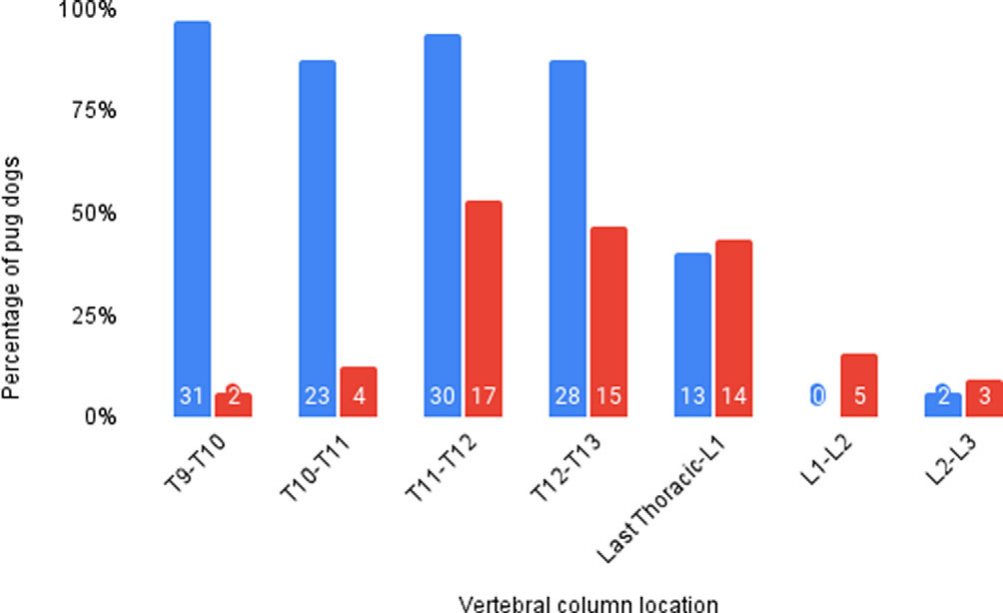
Graphical representation of vertebral segments affected by caudal articular process dysplasia (CAPD) (blue) and myelopathies (red). The discrepancy between the CAPD frequency in the mid‐ to caudal thoracic region, and predominance of non‐CAPD myelopathies in the caudal thoracic to cranial lumbar region, makes an association in pathogenesis less likely. Note that the vertebral segments indicate CAPD of the caudal articular process of the cranial listed vertebra, for example, T9‐T10 refers to caudal articular process of T9. The caudal‐most thoracic vertebra in a few dogs was transitional (with a vestigial rib on 1 side or both sides), we elected to call the caudal‐most joint “last thoracic to L1.”

Thirty‐one of 32 dogs (97%) in the study sample had T3‐L3 myelopathy caused by CM alone, by IVDH alone, by CM in combination with IVDH, or by IVDH in combination with SAD (based on MRI findings) whereas 100% had CAPD (based on CT findings). Of note, in the lumbar region, there was a lower frequency of CAPD relative to a higher percentage of dogs with spinal cord disease (Figure 4).

While 1 dog had a normal spinal cord (based on MRI findings) and CAPD, 19/32 dogs (63%) had multiple spinal cord diseases with accompanying CAPD. There was no apparent relationship between the anatomic locations of CAPD and the anatomic location of the most severe myelopathy or the type of myelopathy within the TL vertebral column.

### 3.3 Pathology

Nineteen of the 32 study dogs (59%) were euthanized or died naturally between December 2014 and March 2019 and were brought to MSU for a nervous system‐focused necropsy. Not all dogs had postmortem examinations; reasons included client distance from the hospital, owner decision, or loss of follow‐up. Eight dogs (25%) were known to have been euthanized or died naturally but no necropsy was performed, and 5 dogs (15.6%) were reported to be alive at the time of last contact (2017‐2020). The age at the time of necropsy ranged from 9.4 to 15.4 years (median 11 years). The time between diagnosis and necropsy ranged from 0 to 1552 days (median 101.5 days). Discounting 2 dogs that were euthanized immediately after MRI, the median interval was 132 days.

**FIGURE 5:**
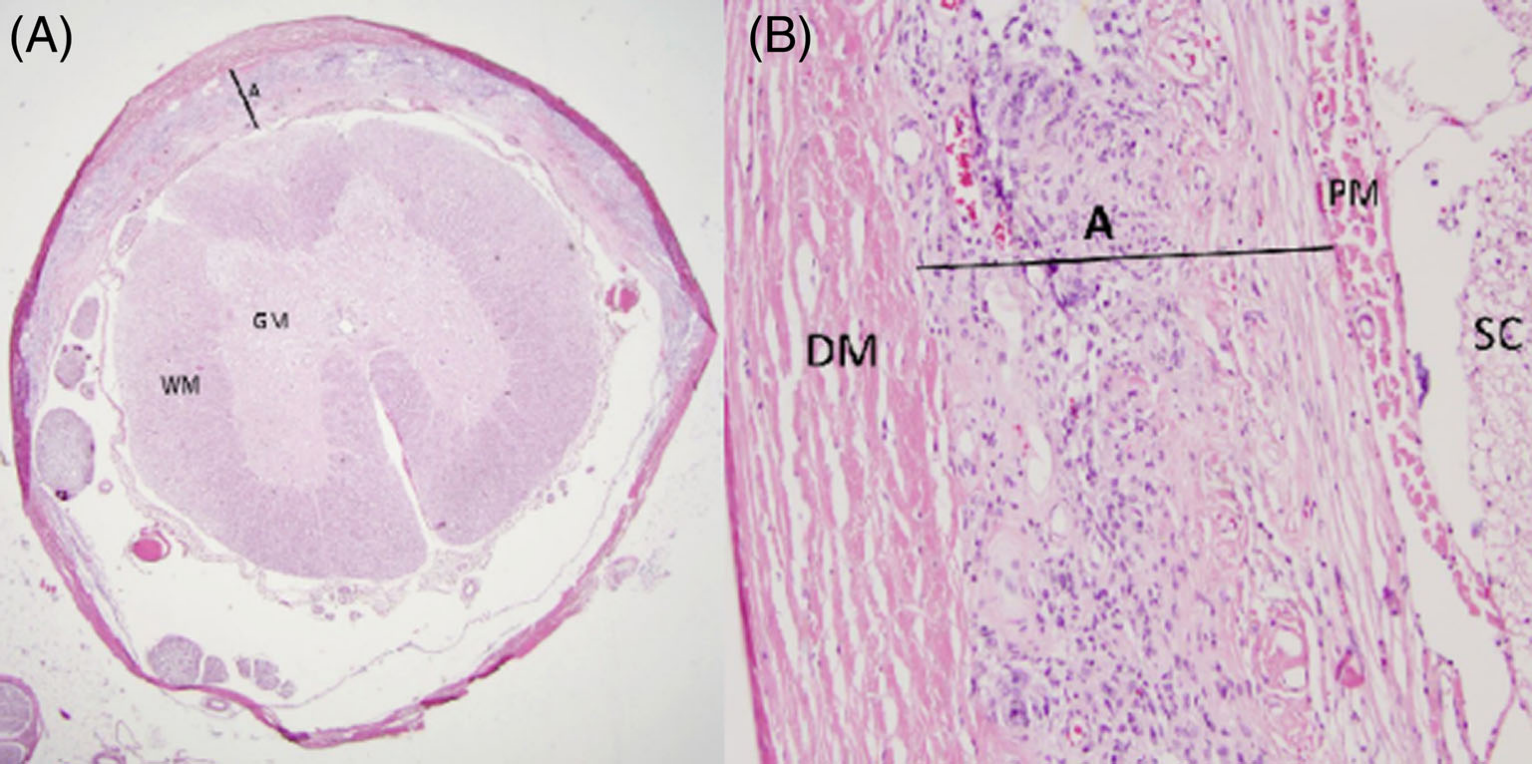
(A) A ×10 photomicrograph of a transverse section of spinal cord stained with hematoxylin and eosin (H&E) displaying a dorsal thickening of the arachnoid layer (A) of the meninges by fibrous tissue. (B) A ×100 photomicrograph of a transverse section of spinal cord stained with H&E demonstrating a detailed look at the arachnoid fibrosis (A). DM, dura mater; GM, gray matter; PM, pia mater; SC, spinal cord; WM, white matter.

In 13 of 19 (68%) necropsied study dogs, IVDH was present at the most severe site of spinal cord compression. Fifteen of 19 (79%) had multifocal IVDH between T3 and L3. On histopathologic examination, 18 (95%) dogs had moderate fibrous thickening of the arachnoid layer of the meninges at 1 or more sites along the SC (Figure 5). For 16 of these 18 dogs (89%), there was arachnoid fibrosis at the most severe site of T3‐L3 myelopathy.

Among the 19 dogs necropsied, 4 (21%) were diagnosed with SAD; 2 of these 4 also had IVDH at the site of the SAD. Spinal arachnoid diverticula were located at T7‐T8, T11‐T12, T12‐T13, and spanning the T10 and T11 vertebrae. These gross findings were generally associated with spinal cord atrophy and axon and myelin degeneration (with or without mild gliosis) histologically. Histopathologically, the dorsal wall of the diverticulum was consistently moderately to markedly thickened because of arachnoid fibrosis (Figure 5); this was appreciable grossly if severe. Additionally, mild chronic arachnoiditis, arachnoid epithelial hyperplasia, whorls of hyalinized collagen, and foci of calcification were noted in some areas. Rarely, fibrosis also involved the dura or pia mater. Also, arachnoid fibrosis was detected with diagnoses of CM, SAD, and IVDH.

Four of the 19 dogs (21%) necropsied had CM only. In these dogs, there was moderate to marked arachnoid fibrosis, which was often apparent grossly. The arachnoid thickening was segmental (usually involving dorsal and lateral funiculi) to fully circumferential, as noted by “A” in Figures 4B and 5A. A consistent histologic feature for CM was axon and myelin degeneration, which usually involved the entire spinal cord white matter in transverse sections. In severe cases, there was spinal cord atrophy, parenchymal collapse, and necrosis, involving both white and gray matter, with obscuration of the distinction between the 2 regions.

## 4 DISCUSSION

Constrictive myelopathy was diagnosed in 20 out of 32 dogs (63%), occurring alone in only 3 pug dogs (9%) and in combination with IVDH in 17 dogs (53%). The majority of pug dogs in this study with T3‐L3 myelopathies had multiple concurrent diagnoses with almost two thirds having more than 1 disease. Computed tomography revealed the presence of CAPD in all pug dogs in this study. There is a high frequency in both clinical and neurologically normal pug dogs ranging between 64% and 97%.^12^, ^14^, ^16^ The high frequency might indicate that pug dogs are genetically predisposed to CAPD, yet results of this study suggest other spinal cord disorders develop over their lifetime, causing signs of neurological disease which might or might not be related to CAPD. In our study sample, the arachnoid layer was found to be affected by fibrosis more than the dura. Although this differs from other reports of CM histopathological findings, this is consistent with other conditions. The overlap of histopathologic findings in CM, SAD, and IVDH in pug dogs might suggest that arachnoid fibrosis‐common in all 3 conditions‐is a reaction peculiar to pug dogs (either familial or inherited) when there is irritation or compression of the spinal cord, regardless of site or diagnosis. A possible breed predilection or genetic predisposition is further suggested by the lack of reports involving other breeds with CM. Constrictive myelopathy might not be a specific diagnosis unto itself, but rather a sequel or contributor to other myelopathies. Also, with every dog included in this study having CAPD, the relationship of CAPD to CM is unclear.

There were no unique clinical signs or clinical courses to favor 1 T3‐L3 myelopathy diagnosis over another. Clinically, there was a general progression toward development of fecal, urinary incontinence, or both within 6 months of initial clinical signs. Spinal cord lesions were most frequent in the T11‐L1 vertebral region, regardless of site(s) of vertebral CAPD. Constrictive myelopathy with additional diagnoses was the most common diagnosis category based on MRI findings. None of the studied pug dogs diagnosed with CM had hyperesthesia noted on clinical examination. This is consistent with previous reports of CM.^2^

Almost all pug dogs in this study had multiple sites of spinal cord compression at necropsy, with a majority having multiple diagnoses between T3‐L3 (IVDH, SAD, and CM). Therefore, making a definitive diagnosis in pug dogs with T3‐L3 myelopathies can be difficult not only for the figure clinician but for the pathologist. This study highlights multiple disease occurrences and the difficulty in determining the main cause of a pug dog's paraparesis, even with the aid of MRI. The results of necropsy findings can be used to develop a better understanding of the MRI interpretations of lesions.

Limitations within this study included a lack of neurologically normal control pug dogs, preventing a determination of potential subclinical myelopathies as well as an assessment of CAPD occurrence in normal pug dogs. As CAPD might be present in a majority of pug dogs and our study examined only neurologically abnormal dogs, true prevalence of the condition in the breed remains unknown. Additionally, DNA testing for genetic mutations causing degenerative myelopathy (DM) was not a part of this study; it is possible that DM was yet another diagnosis to consider in these dogs with chronic progressive T3‐L3 myelopathies. However, clinical cases of DM in pug dogs are very rare with but 1 report appearing in literature.^17^ Importantly, the diagnostic imaging criteria for a CM diagnosis has not been well described at this time; as more is learned about this condition, specific diagnostic criteria may come into sharper focus.

This study analyzed the frequencies of common T3‐L3 myelopathies in pug dogs, and attempted to correlate vertebral abnormalities and spinal cord diseases. While CAPD was present in 100% of our study dogs, and at several thoracic vertebral sites in each individual animal, the location of the vertebral abnormality was not necessarily associated with the sites of spinal cord disease. This might suggest that CAPD of itself is clinically unremarkable.

## CONFLICT OF INTEREST DECLARATION

Authors declare no conflict of interest.

## OFF‐LABEL ANTIMICROBIAL DECLARATION

Authors declare no off‐label use of antimicrobials.

## INSTITUTIONAL ANIMAL CARE AND USE COMMITTEE (IACUC) OR OTHER APPROVAL DECLARATION

Approved by the Michigan State University IACUC before enrollment of dogs (protocol 08/14‐137‐00).

## HUMAN ETHICS APPROVAL DECLARATION

Authors declare human ethics approval was not needed for this study.
